# Research on the Prediction of A-Share “High Stock Dividend” Phenomenon—A Feature Adaptive Improved Multi-Layers Ensemble Model

**DOI:** 10.3390/e23040416

**Published:** 2021-03-31

**Authors:** Yi Fu, Bingwen Li, Jinshi Zhao, Qianwen Bi

**Affiliations:** 1 School of Finance and Business, Shanghai Normal University, Shanghai 200234, China; fuyi@shnu.edu.cn (Y.F.); 1000478437@smail.shnu.edu.cn (B.L.); zjs@shnu.edu.cn (J.Z.); 2 Woodbury Business School, Department of Finance and Economics, Utah Valley University, Orem, UT 84058, USA

**Keywords:** high stock dividend, financial market, feature adaptive improvement, multi-layers stacking algorithm

## Abstract

Since the “high stock dividend” of A-share companies in China often leads to the short-term stock price increase, this phenomenon’s prediction has been widely concerned by academia and industry. In this study, a new multi-layer stacking ensemble algorithm is proposed. Unlike the classic stacking ensemble algorithm that focused on the differentiation of base models, this paper used the equal weight comprehensive feature evaluation method to select features before predicting the base model and used a genetic algorithm to match the optimal feature subset for each base model. After the base model’s output prediction, the LightGBM (LGB) model was added to the algorithm as a secondary information extraction layer. Finally, the algorithm inputs the extracted information into the Logistic Regression (LR) model to complete the prediction of the “high stock dividend” phenomenon. Using the A-share market data from 2010 to 2019 for simulation and evaluation, the proposed model improves the AUC (Area Under Curve) and F1 score by 0.173 and 0.303, respectively, compared to the baseline model. The prediction results shed light on event-driven investment strategies.

## 1. Introduction

The rapidly growing securities market in China drives the participation and curiosity of investors. Investors’ reaction on information plays a central role in modern financial markets globally. As one of the key signals of a company’s profitability and sustainability, Dividend policy becomes an essential trigger of stock price movements. Despite what the Modigliani-Miller theorem states [1], the effects of dividend policies are puzzling, especially in China.

China’s A-share market is an important global investment market. As of 1 December 2020, the total market value of China’s A-share listed companies in Shenzhen and Shanghai stocks reached 82.92 trillion yuan. In the global market, the total market value of A-share listed companies is second only to the United States, ranking second globally [2]. Unlike other listed companies’ emphasis on cash dividends, there is a long-term phenomenon in China’s A-share market, called a “high stock dividend”. For every ten shares of holding, the company will transfer five shares or more to shareholders [3], and its publication time is often concentrated in the annual report period. On 4 April 2018, the Shanghai Stock Exchange and the Shenzhen Stock Exchange announced the guidelines for disclosing listed companies’ high stock dividend information (Draft). This disclosure imposed strict restrictions on the reduction and sales of shares held by relevant shareholders, the company’s net profit, and the company’s earnings per share, accelerating the resulting downward trend in the number of high stock dividend distribution which began in 2016. Some research shows that the stock dividend announcements have a positive impact. In contrast, the cash dividend announcements negatively impact abnormal returns for Chinese companies [4], and the intensity of high stock dividends is positively correlated with the scale of significant shareholders’ reduction [5]. Part insiders may consciously take advantage of investors’ irrational preferences to achieve their rational self-interest motivations like stock sales in high stock dividends. High stock dividends have the feature of an instrument [6]. Therefore, the prediction of stock dividend distribution is meaningful for alleviating the information asymmetry in the investment market, assisting investors in making investment decisions, and providing a decision-making basis for the market supervision department. That explains why a more accurate prediction on this particular phenomenon is valuable.

This study proposes a feature adaptive improved multi-layers ensemble model to boost prediction accuracy of “high stock dividends”. The following structure of this paper is as follows. In Section 2, the previous results related to this study are reviewed, and the main contributions of stacking ensemble algorithm improving are also introduced. Section 3 describes each part of the feature adaptive improved multi-layers ensemble model in detail, including the optimization of feature engineering, the adaptive matching of the base model and the feature subset, and the design of the second feature extraction layer. All model backtesting results with A-share historical data are analyzed in Section 4 to evaluate the model’s predictive ability. Finally, the research is summarized, and the follow-up work is presented in Section 5.

## 2. Literature Review

The previous studies discussed the phenomenon of high stock dividends mainly included three aspects: motivation, excess return, and prediction methods.

In the existing literature, there are several different views on the phenomenon. Most of the work was based on the aspects of traditional economics and behavioral economics. Under the traditional economics framework, some scholars analyzed the high stock dividend phenomenon from the perspective of the signaling theory [7] and believed that the company’s management intended to pass the information of the company’s future performance to the investors through dividend policy. Many scholars demonstrated the effect of dividend policy on transmitting positive signals to the company’s operation [8,9,10]. According to the optimal price theory put forward by other scholars, the excessive stock price will demand that small and medium-sized investors have more capital, which will restrict their trading behaviors. Split shares or dividend policies can reduce the stock price and improve liquidity, making the stock price be in a more reasonable range [11,12,13]. In behavioral finance, a class of views supported the dividend catering theory, pointing out that when investors had irrational preferences, the company’s management had an incentive to cater to investors for proposing related dividend policies. Another group of researchers believed in price illusion theory, pointing out that nominal price changes due to stock dividend distribution can affect investors’ decision-making [14].

Some scholars’ research focused on the excess return causing by high stock dividend. Eng et al. (2014) empirically found that the strengthening of stock split supervision would reduce the information asymmetry [15], which made the return on the announcement day shift from high correlation with lagging profitability of the previous financial report to high correlation with future profitability. Furthermore, as Huang and Paul (2017) pointed out [16], institutional investors preferred companies that paid dividends. There was often an inevitable excess return in the A-share market before and after the occurrence of a high stock dividend phenomenon [17]. Therefore, the successful prediction of this phenomenon will help investors to build an effective event-driven strategy and obtain excess returns.

Another kind of research focused on the prediction of the stock dividends, but this kind of research was relatively unpopular. Ezell and Rubiales (1975) firstly used the idea of discrete dependent variable modeling to study the dividend policy prediction [18]. Bae (2010) introduced decision tree, multi-layer perceptron, and support vector machine (SVM) models [19]. Taking the data of Korean listed companies as an example, Bae found that the SVM model based on RBF kernel could accurately predict the dividend policy of South Korea. Xiong et al. (2012) used the logistic regression model to predict the high stock dividend phenomenon from 2007 to 2011 [20]. Multi-layers perceptron was proposed by Dong and Zhao (2019) to predict the phenomenon of the high stock dividend distribution, which improved the accuracy rate by 12% based on the logistic regression model [21].

According to the previous studies, there are still two aspects that should be improved: (1) Classical methods usually choose one method for feature selection. As we know, the selection principles of different single feature selection methods are different. As a result, the feature sets obtained by different methods are often different. In other words, some features can be selected by one method, but at the same time, they will be missed by another method. The single feature screening method has a specific feature omission risk. (2) Single-method models have strong prediction ability but relatively low generalization ability. Therefore, some studies use the stacking algorithm to integrate the base model’s output and improve the generalization ability. In this general stacking algorithm, the base model’s output is usually weighted or used as the input of a classification model to predict the final result. However, such a method lacks information extraction for the output of the base model, which limits the use efficiency of the feature information of the model and restricts the model’s predictive ability.

This paper proposes a feature adaptive improved multi-layers ensemble model, an improved stacking ensemble model. This study’s main contributions are as follows: (1) We use the equal weight feature comprehensive evaluation method to select the effective features. This method can take advantage of various single feature selection methods and reduce the risk of missing essential features. (2) Genetic algorithm is used to customize the optimal feature subset for each base model to improve each base model’s predictive ability, which is the basis for improving the overall predictive ability of the model. (3) With the inspiration of the deep tree model [22], this paper uses the GBDT (Gradient Boosting Decision Tree) [23] model as the feature information extraction layer of the base model output in the stacking algorithm [24]. The base model output is mapped to the new space to achieve new features and use the new feature to make predictions through feature information extraction. This work improves the prediction accuracy of the model.

## 3. The Design of Feature Adaptive Improved Multi-Layers Ensemble Model

After summarizing the relevant literature, this section will discuss the design of the feature adaptive improved multi-layers ensemble model. The modeling process is divided into three parts: feature engineering, construction and selection of feature adaptive base models, and the multi-layer ensemble model. 

This paper investigates the prediction of the “High Stock Dividend” phenomenon. Through identification of the A-listed companies with high stock dividend in the next six months as “1”, otherwise as “0”, the prediction observation can be transformed into a binary variable. Previous studies showed machine learning is effective when solving this kind of question, such as the rise and fall of stocks, debt default, etc. [25,26]. Feature selection plays an important role in machine learning prediction, and appropriate features can greatly improve the prediction ability of machine learning methods [27]. There are two common feature selection methods [28]: univariate methods (such as F value [29], maximum information coefficient (MIC) value [30], information value (IV) value [31], etc.) and multivariate methods (such as recurrent feature elimination (RFE) [32], etc.). All feature selection methods are based on a specific correlation or importance measurement method, but the relationship between variables is usually complex. Different feature selection methods may get different subsets [33]. Some variables may be tail features in one method and head features in another, which means that univariate methods exist the risk of missing important features. For this reason, the ensemble feature selection method will be used in this model.

Since the single-method model is weak with generalization ability [34], we decide to use the stacking ensemble model in this study. Stacking framework has been used in machine learning applications in different fields [35,36]. The idea of the framework is mainly divided into two parts. The first part integrates the first several layers of the model to achieve the generalization ability of the model as much as possible, and the second part integrates all the information and improving the robustness of the last layer. Due to the fact that the principles of different sub-models are different, their requirements for features may also be different to a certain degree. However, previous studies usually train and integrate different sub-models with the same selected feature dataset [37], which makes some sub-models lack the input of important features in training, and it is difficult for sub-models to achieve optimal performance and ultimately affect the prediction ability of stacking method. To improve the performance of the stacking framework, based on feature selection, this paper will use a genetic algorithm [38] to find the optimal feature subset of the corresponding model and train the model independently to achieve the consistency between the base model and the feature subset. The output of each base model can be regarded as a newly generated feature. To improve the efficiency of information utilization, we then need to cross these output features and extract new features. As an essential branch of machine learning, the tree model originated from the ID3 algorithm in 1986. After decades of development, tree models with good performance, such as CART (Classification and Regression Tree), C5.0, and others, have been proposed, making the tree model very popular. The tree model’s basic metrics include Gini impurity, Information gain, etc., which are based on the concept of entropy and information theory, which makes the tree model less demanding on the amount of data compared to other models. Because of this advantage, the tree model is well suited to act as the second feature extraction layer in “high stock dividend” prediction. The GBDT model is selected to generate new features from the base model’s output based on the feature cross-ability. Finally, Logistic Regression (LR) model is used to extract information from these features generated at the last level and outputs the final prediction results, which will improve the model’s generation ability.

**Figure 1:**
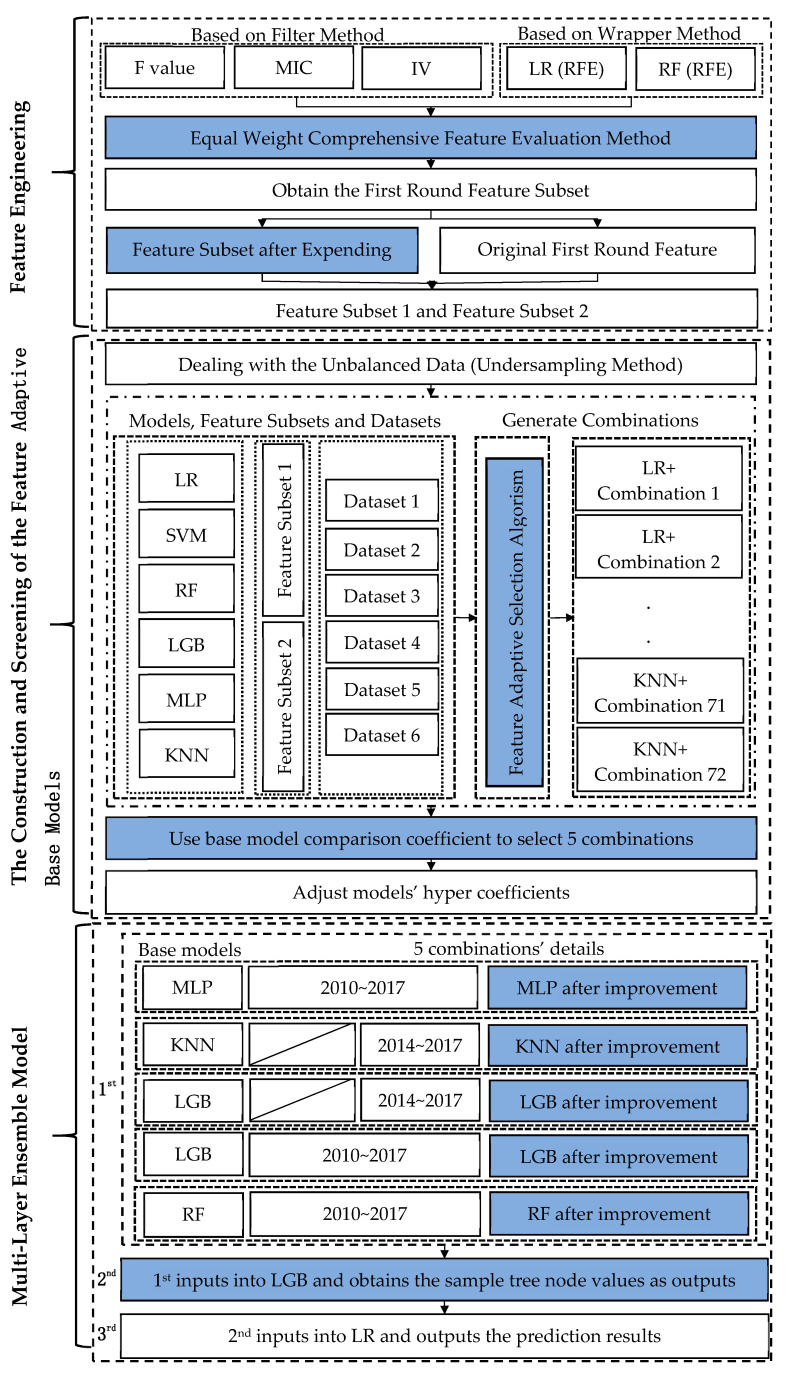
Technical path (1st represents the first layer, 2nd represents the second layer and 3rd represents the third layer; after improvement means after feature adaptive selection algorism improves the original feature subsets).

As shown in Figure 1, the first part of the model is feature engineering. In this part, the equal weight comprehensive feature evaluation method is used to find out the features related to high stock dividends, and the corresponding feature subset 1 is obtained. The feature subset 2 is then obtained by automatically expanding the feature subset 1 by the genetic programming method. The second part of the model is the construction and selection of the feature adaptive base model. In this paper, we use LR [39], SVM [40], Random Forest (RF) [41], LightGBM (LGB) [42], Multi-Layers Perceptron (MLP) [43], and K-Nearest Neighbor (KNN) [44] models with multiple datasets and feature subset combinations using feature adaptive selection algorism to form the feature adaptive base model. According to the base model comparison coefficient (formula 1) of the base models [45], the base model with better performance and differentiated output results in the verification set in 2018 is selected. The specific steps are: (1) Calculate the numerator, which is the AUC [46] of each base model in the validation dataset. (2) Select the model having the highest AUC as the target model. (3) Calculate the pearson correlation coefficient between the AUC of the target model and the AUC of the other base models. (4) Use the formula (1) to obtain the base model comparison coefficient, which will be used as the metrics to select the base models.

(1) $$base\;model\;comparison\;coefficient=\frac{\;AUC\;of\;validation\;dataset}{output\;Pearson\;correlation\;among\;the\;base\;models}$$

The last part of the model is the construction of a multi-layer ensemble model. In this paper, a multi-layer stacking ensemble model is designed to further improve the prediction and generalization ability based on the base models. Each part will be described in detail below.

### 3.1. Feature Engineering

The goal of feature engineering is to screen features in all directions (from the perspective of a linear relationship, nonlinear relationship, and model performance) without loss of model accuracy (AUC). The specific steps are as follows: (1) For single-factor analysis, using one-way ANOVA (F value) to investigate the linear relationship between features and target variables, and using family-wise error rate (FWE) error measure methods to investigate whether the features suitable under this inspection. (2) The maximum information coefficient (MIC) is used to investigate the arbitrary statistical relationship between features and target variables. The MIC value was scored with a fixed proportion (more than 50% quantile). (3) Firstly, the genetic algorithm is used to divide the features into boxes. The information value (IV) is used to check whether the features suitable under this inspection (more than 50% quantile). (4) Recursive feature elimination (RFE) with cross-validation was used to investigate the linear model’s importance and nonlinear model features by the LR model and the RF model with the L1 regular term. According to the output of RFE, whether the features scored under the inspection was evaluated (set to retain 50% features). After the above feature screening, each feature gets 5 groups of scores (1 point for each group). Finally, the final score of each feature is obtained by using the equal weight method. If the score is more than 4(including 4), it will be in the feature subset 1 with 48 features. Secondly, this paper uses genetic programming to mine features, which can automatically discover the potential relationship of features and get feature subset 2 with 100 features.

Considering the characteristics of the high stock dividend prediction problem, the model’s core evaluation indicators are determined as AUC and F1 score [47]. On the one hand, the key index of the prediction is AUC, which comprehensively considers the positive and negative examples and reflects the degree of fitting of the model, which is suitable for the unbalanced two classification problem in this paper. However, the F1 score can comprehensively reflect the model’s accuracy and recall rate and comprehensively reflect its prediction ability.

### 3.2. The Construction and Screening of the Feature Adaptive Base Models

**Figure 2:**
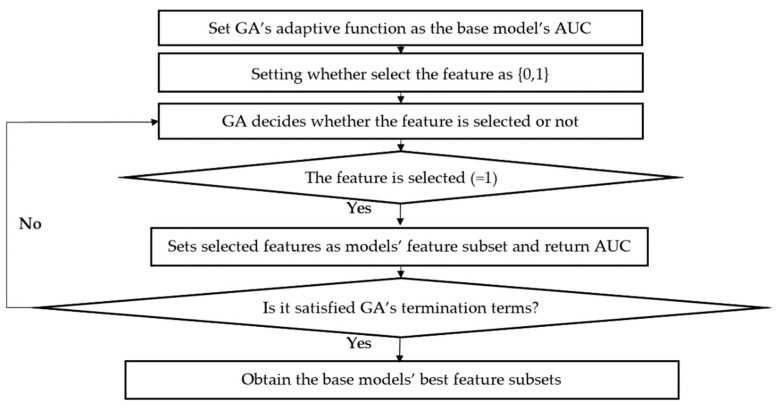
Flow chart of the adaptive feature selection algorithm.

Based on the particle swarm optimization (PSO) feature selection algorithm proposed by Dai and Li [48], this paper presents an adaptive feature selection algorithm. Considering that the RFE method only selects features from the perspective of feature importance, it does not take into account the promotion of feature subset on the model’s prediction ability. In this paper, the AUC returned by each base model is used as the adaptive function to be optimized, and the feature selection model is designed by using a genetic algorithm. The specific algorithm flow is shown in Figure 2.

**Figure 3:**
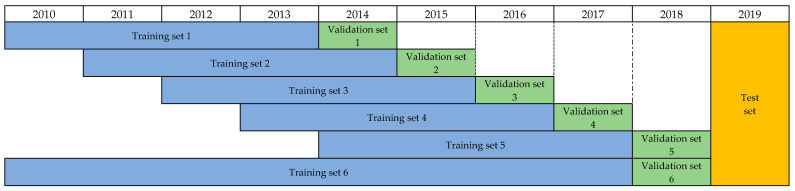
Constructing training set by sliding window method.

After the design of the adaptive feature selection algorithm, the model uses the instance hardness threshold to process the unbalanced data; LR, SVM, RF, LGB, MLP, and KNN are selected as the base models to be selected, and six sets of datasets are constructed with the sliding window method in Figure 3. Then, 72 combinations (6×2×6=72) of different models, feature subsets, and datasets are combined with the adaptive feature improvement method to find the corresponding optimal feature subset (AUC is calculated by verification set when the algorithm is applied). Finally, taking 2018′s data as the verification set, the AUC of each combination is obtained, and the formula (1) is constructed (the model with the highest AUC is taken as a reference base).

### 3.3. Multi-Layer Ensemble Model

Based on the construction idea of the comparison coefficient of the base model (in the first layer of Figure 1), this paper has screened out the basic model with strong expressive ability and a certain degree of difference. Because the corresponding dataset length and the feature subset are different, the model adopts a stacking framework to express each model’s advantages. For the second layer design of Figure 1, traditional ensemble ideas often need the same length of datasets because of the different lengths. Simultaneously, some base models often fail to have the best “memory” ability, performance, and difference on the same dataset. Therefore, the model adopts the GBDT feature derivation framework and uses the LGB model to extract features further. The LGB model is used as the second layer of the stacking ensemble model, while the sample tree node information of LGB is extracted as the output of the second layer after the input of the predicted value of the base models. In the third layer of Figure 1, all samples’ tree node information is used as the input of the LR model who has good robustness. The multi-layer stacking ensemble model integrates various datasets and feature subsets and uses the idea of deep learning to improve the prediction ability based on multiple strong learners. The memory ability of the machine learning model is explored as much as possible in the first layer. Then, the model’s generalization ability is improved by the second layer, and the risk of model over-fitting is reduced by using the third layer.

## 4. Results on the Test and Evaluation of High Stock Dividend Prediction Model

According to the modeling process in Figure 1, this section will test and evaluate the model (win10 + python3.7). The test and evaluation of the model include the following three parts: the feature screening evaluation of the equal weight feature comprehensive evaluation method; the testing and evaluation of the multi-layer ensemble model under the adaptive feature selection method; and the overall prediction evaluation of the adaptive improved multi-layer ensemble model.

### 4.1. Data Preprocessing and Feature Engineering

The data in this paper are from RESSET financial database. The features are the third-quarter financial report data of China’s A-share companies from 2010 to 2019 and the price volume data corresponding to the first working day of November of the corresponding financial year. The prediction target is the corresponding high stock dividend rate (high stock dividend rate is greater or equal to 0.5) corresponding to 2011–2020 (published in 2010–2019 annual report). In this paper, 245 features are divided into 13 categories. For the obtained samples, the specific data preprocessing scheme of the model is shown in Table 1. The sample size after data preprocessing is 19,753, and the number of effective characteristics is 219, which is shown in Table 2.

After data preprocessing, the following will be the test and evaluation of feature engineering:

Firstly, this paper investigates the linear relationship between each feature and whether the company will issue a high stock dividend or not by F value and finds the arbitrary statistical relationship by MIC. Secondly, because the original continuous data may have considerable noise, which may affect the model, this paper introduces the IV feature selection method after the optimal box division of the genetic algorithm to investigate the features’ performance. This paper uses a genetic algorithm to divide all features into ten boxes with the maximum IV value as the adaptive function due to the subjectivity and lack of clear mathematical meaning in the traditional discretization. According to the features’ IV value, the features are screened, and the top 50% of the feature are obtained. Finally, LR with the L1 regular term and RF models are used as the base models of the RFE method. If RFE selects it, the feature will get a score.

Through the above five steps, five groups of scores are obtained. The features with a score of four or five are selected as feature subset 1. The specific selection solution is shown in Table 3. It can be seen from Table 3 that most of the categories have features been selected, while profitability, operation capacity, income quality, DuPont analysis, and industry information are not selected. In addition, genetic programming is used to extend feature subset 1 to 100 as feature subset 2.

### 4.2. The Evaluation of High Stock Dividend Prediction Model

This paper will first deal with data imbalance based on the two feature subsets and six datasets obtained above. Then we will select the base model of adaptive improved stacking ensemble algorithm. Finally, we will build a three-layer stacking ensemble model while showing and comparing the results.

In this paper, the prediction of high stock dividends needs to deal with unbalanced data before modeling. The attempts made in this paper for unbalanced data are shown in Appendix Table A1. Due to the limitation of computing power, this paper only uses all 219 features, 2010–2013 as the training set (2014 as the test set), and the LGB model as the learner to compare the relative performance of unbalanced data sampling methods (some sampling methods based on nearest neighbor algorithm have high time complexity, so this paper ignores them). In the comprehensive comparison, because the instance hardness threshold method has the highest AUC value, it is more suitable for this dataset. The following will show the screening results of the feature adaptive base model and a multi-layer ensemble model.

#### 4.2.1. Screening Results of Feature Adaptive Base Models

As described in Figure 1, the method for constructing the base model of adaptive improved stacking algorithm has two feature subsets, six datasets, and six models, which gets a total of 72 base model combinations. Constructing different datasets and different feature subsets lets the base models learn different information about the dataset as much as possible. Table 4 below shows the number of original features under each combination, the number of features after adaptive feature filtering, and AUC changes under the combination of two feature subsets (see Appendix Table A2 for the complete results).

After testing and comparing each base model’s original verification set, this paper uses the 2018 data as the verification set to calculate the AUC of the verification set. The model selects feature subset 1 in dataset six and takes the MLP model as a reference base, calculates the base model’s comparison coefficient according to formula (1) above, and then obtains five base models of stacking layer 1. Most of the base models in this paper meet the two requirements of stacking base model selection: the diversity of the basic model principle and good prediction ability. Finally, this paper adjusts the hyperparameters using fivefold cross-validation on the selected base model’s original training set. The results are shown in Table 5. As can be seen from Table 5, except for the performance of LGB under dataset six after parameter adjustment, other models have improved to some extent.

#### 4.2.2. The Results of Multi-Layer Ensemble Model

This paper constructs the second and third layers of stacking based on the idea of stacking ensemble and GBDT feature derivation. Specifically, after getting five groups of new features output from the five base models, this paper inputs them into the LGB model and obtains the information of the location of the sample in the leaf node of the tree (because the LGB can accommodate missing values and does not affect the construction of the decision tree, though the datasets of the base models in this paper are different). The LR model with lower complexity is adopted after obtaining the high-dimensional sparse new features of the second layer. After that, the results of this paper on the test set (2019) are obtained (see Table 3 for the results of layer two and layer three tunings). Finally, as compared with the results in Table 6, the AUC and F1 scores of the adaptive improved three-layer stacking integration model are improved by 0.173 and 0.303 respectively compared to the baseline model, which is better than the results of all the base models.

The result of backtesting and comparison shows that the model has good predictive capabilities. Compared with previous models, forecasting ability mainly comes from the following three aspects of model improvement. First of all, the equal weight comprehensive feature evaluation method is used to consider the differences in various feature selection methods and effectively avoid the omission of effective features. For example, some price volume variables are not selected under the F value method but are selected under the MIC value method. Secondly, the adaptive feature evaluation method customizes different feature subsets for different base models, which can avoid the mismatch between features and base models, improve the predictive capabilities of each base model, and output high-quality information sources for the final information integration. Finally, a multi-layer ensemble model is built for further automatic feature extraction and abstraction of the output from the base models. This work improves the generalization ability of the model. As the model’s final output layer, the LR model integrates information and predicts the high stock dividend phenomenon.

Due to the lack of definite standards, it is difficult for us to determine an optimal model at the beginning stage of modeling. The idea of the ensemble model has become a popular machine learning solution. However, it is worth mentioning that in the process of model integration, we need to make each base model achieve the best prediction ability on its own. There are many aspects to the optimization of the base model. A significant one is to customize the corresponding feature subset for each base model so that different base models can effectively extract the information of each feature variable. The experimental results of this study also support the validity of this idea.

There is no doubt that any prediction on the stock market by nature is crucial and valuable. However, it is not an easy task. Variables to be considered but not limited to reflect a long list: microeconomic and macroeconomic factors, financial statements, market conditions, regulatory policies, and individuals’ sentimental behavior. As the world is advancing, algorithm models using machine learning are applied to the prediction game. The improved prediction accuracy provides insights to investors and policymakers.

All the results in this study are based on historical data backtesting, and there are still differences from the real market environment. Simultaneously, due to the limitation of the existing data feature dimensions, the feature input of the model in this paper still needs to be continuously improved, which is also the focus of our future work.

## 5. Conclusions

In this paper, based on equal weight comprehensive feature evaluation, GBDT, and stacking framework, the high stock dividend phenomenon’s existing prediction models are improved. A feature adaptive improved multi-layers ensemble model is proposed. This paper’s main contributions are as follows: (1) For the prediction of the high stock dividend phenomenon, the multi-layer stacking ensemble model constructed in this paper can predict the high stock dividend phenomenon accurately. Compared with the baseline model, the AUC is improved by 0.173, and the F1 score is increased by 0.303. (2) A complete comprehensive feature evaluation method and a model-based feature adaptive selection algorithm are proposed that can effectively select the feature subset which is more suitable for the corresponding model. (3) This paper proposes a multi-layer stacking ensemble model design, which can integrate models of different length datasets and feature subsets.

This paper’s practical significance is as follows: (1) From the investment perspective, this paper provides better prediction results than the existing methods, helping institutional investors better construct event-driven investment strategies on the high stock dividend issue. (2) From the perspective of policy, the existing policies are based on the previous scholars’ interpretation of the motivation behind the phenomenon of the high cash dividend. With the help of this paper’s high accuracy prediction model, regulators can conduct qualification screening for companies that may have a high stock dividend policy in the following year from November every year. 

Although the model proposed in this paper has good prediction ability, there are still some limitations in this research, which will be possible future research directions. Firstly, this study uses the equal weight comprehensive feature evaluation method to filter the features that predict the “high stock dividend” phenomenon. Selected features increase the model’s information input consistency, but their interpretability is not provided. Feature interpretation under the stacking framework will be one of our future research works. Secondly, because the “high stock dividend” dataset is highly unbalanced in the securities market, the number of “high stock dividend” listed companies is far fewer than other listed companies. This current situation limits the predictive ability of the model. Some sampling methods have been used in this study’s data processing and have played some role in the improvement of model training. However, the research of this problem still requires better data balancing processing methods. Studying the sample structure of unbalanced data is also our future research agenda.

## Figures and Tables

**Table 1:** Data preprocessing.

Step	Raw Data Issues	Corresponding Solutions
1	Some samples’ data may be questionable	Excluding ST and ST* stocks
2	Missing values in some features	Features with missing values > 50% are eliminated
3	Missing values in some samples	Excluding the samples of banking industry
4	Severe extreme value issues	Use percentile method to remove the extreme values
5	Serious data scales issues	The original data were standardized by min-max feature scaling

ST and ST* mean the stock is a warned status by the China Securities Regulatory Commission.

**Table 2:** Data overview after preprocessing.

Category	Category ID	Original Feature Num.	Missing Value ≥ 50%	Final Feature Num.
Target variable	Y	1	0	1
Per share variable	X1	21	1	20
Profitability	X2	40	1	39
Solvency	X3	17	0	17
Ability to grow	X4	20	0	20
Operational capacity	X5	11	0	11
Cash flow variable	X6	20	5	15
Capital structure	X7	16	2	14
Revenue quality	X8	10	2	8
DuPont analysis	X9	6	0	6
Income statement items	X10	16	3	13
Assets and liabilities	X11	38	12	16
Price volume variable	X12	29	0	29
Industry category	X13	1	0	1

**Table 3:** The selected result of each method.

	Category	Filter Method			Wrapper Method		Screening Result	
		F Value	MIC	IV	RFE(L1)	RFE(RF)	Num.	Proportion
Per share variable	X1	80.00%	40.00%	40.00%	60.00%	70.00%	7	35.00%
Profitability	X2	92.31%	15.38%	33.33%	12.82%	0.00%	0	0.00%
Solvency	X3	64.71%	82.35%	52.94%	0.24%	11.76%	2	5.13%
Ability to grow	X4	40.00%	60.00%	50.00%	60.00%	25.00%	6	35.29%
Operational capacity	X5	36.36%	0.00%	36.36%	45.45%	0.00%	0	0.00%
Cash flow variable	X6	80.00%	26.67%	40.00%	46.67%	20.00%	3	15.00%
Capital structure	X7	100.00%	50.00%	21.43%	21.43%	7.14%	1	9.09%
Revenue quality	X8	50.00%	12.50%	37.50%	50.00%	0.00%	0	0.00%
DuPont analysis	X9	66.67%	0.00%	50.00%	16.67%	0.00%	0	0.00%
Income statement items	X10	100.00%	53.85%	46.15%	30.77%	15.38%	2	13.33%
Assets and liabilities	X11	100.00%	92.31%	53.85%	69.23%	65.38%	17	65.38%
Price volume variable	X12	75.86%	58.62%	58.62%	65.52%	58.62%	10	34.48%
Industry category	X13	100.00%	0.00%	0.00%	0.00%	0.00%	0	0.00%

**Table 4:** The result of the feature adaptive base models

Feature Subsets	Datasets	Before or after Improvement	AUC	Before or after Improvement	AUC	Before or after Improvement	AUC
		LR		SVM		RF	
1	1	Before	0.666	After	0.659	After	0.764
2	1	Before	0.762	After	0.745	After	0.760
		LGB		MLP		KNN	
1	1	After	0.749	After	0.782	After	0.767
2	1	Before	0.771	Before	0.750	After	0.756
		LR		SVM		RF	
1	2	After	0.674	After	0.753	After	0.807
2	2	Before	0.844	After	0.845	After	0.808
		LGB		MLP		KNN	
1	2	After	0.753	After	0.788	After	0.766
2	2	After	0.848	After	0.854	After	0.803
		LR		SVM		RF	
1	3	After	0.692	After	0.667	After	0.863
2	3	After	0.902	After	0.870	After	0.887
		LGB		MLP		KNN	
1	3	After	0.878	After	0.849	After	0.862
2	3	After	0.886	After	0.886	Before	0.904
		LR		SVM		RF	
1	4	Before	0.713	After	0.690	Before	0.715
2	4	After	0.585	After	0.574	After	0.602
		LGB		MLP		KNN	
1	4	Before	0.721	After	0.714	After	0.714
2	4	Before	0.609	After	0.660	After	0.587
		LR		SVM		RF	
1	5	After	0.679	After	0.668	After	0.700
2	5	After	0.662	After	0.578	Before	0.707
		LGB		MLP		KNN	
1	5	Before	0.705	After	0.698	After	0.706
2	5	Before	0.682	After	0.684	Before	0.715
		LR		SVM		RF	
1	6	Before	0.683	After	0.663	Before	0.703
2	6	Before	0.644	After	0.554	Before	0.677
		LGB		MLP		KNN	
1	6	After	0.724	After	0.725	After	0.711
2	6	Before	0.689	After	0.644	Before	0.707

a. AUC here is comparable only under the same dataset. Due to the strict policies of high stock dividend in early 2018, the AUC of model under datasets 4, 5, and 6 decreased significantly. b. “Before” means that the features of the input model have not been filtered by the adaptive feature selection method; “After” means that the features of the input model have been filtered by the adaptive feature selection method. The process can be found in Figure 2.

**Table 5:** The comparation of the base models’ performance before and after the adjustment of the hyper parameters.

Model	Feature Subset	Dataset	Before or after Improvement	Base Model Comparison Coefficient	AUC	
					After	Before
MLP	Feature subset1	Dataset 6	After	/	0.760	0.725
KNN	Feature subset1	Dataset 5	After	0.919	0.724	0.706
LGB	Feature subset1	Dataset 5	After	0.918	0.721	0.705
LGB	Feature subset1	Dataset 6	After	0.903	0.709	0.724
RF	Feature subset1	Dataset 6	After	0.891	0.736	0.703

a. “Before” means that AUC comes from a model without parameter optimization; “After” means that AUC comes from a model with parameter optimization. b. The validation dataset of all models here is the data of 2018. Using the same data set as the validation set can help us compare the AUC of the model under the same experimental environment (data), so that the comparison result is reliable (Dataset division can be seen in Figure 3). c. The hyper parameters are shown in Appendix Table A3.

**Table 6:** The comparation of the results of adaptive multi-layer ensemble model, base models and baseline models.

Model	Feature Subset	Dataset	Before or after Improvement	AUC	F1 Score
LR (baseline)	/			0.594	0.268
Base model 1 (MLP)	Feature subset1	Dataset 6	After	0.760	0.489
Base model 2 (KNN)	Feature subset1	Dataset 5	After	0.724	0.466
Base model 3 (LGB)	Feature subset1	Dataset 5	After	0.721	0.448
Base model 4 (LGB)	Feature subset1	Dataset 6	After	0.709	0.437
Base model 5 (RF)	Feature subset1	Dataset 6	After	0.736	0.459
Adaptive Multi-Layer Ensemble Model	/			0.767	0.571

## Data Availability

Data was obtained from RESSET Database and is available for registered users from the URL: http://db.resset.com/ (accessed on 5 September 2020).

## Appendix A

**Table A1:** The comparation of the result of the methods dealing with the unbalanced data.

	Sampling Method	AUC
Under sampling	under_sampling.EditedNearestNeighbours	0.586
	under_sampling.RepeatedEditedNearestNeighbours	0.644
	under_sampling.AllKNN	0.604
	under_sampling.InstanceHardnessThreshold	0.776
	under_sampling.NearMiss	0.649
	under_sampling.NeighbourhoodCleaningRule	0.583
	under_sampling.OneSidedSelection	0.531
	under_sampling.RandomUnderSampler	0.625
	under_sampling.TomekLinks	0.514
Over sampling	over_sampling.ADASYN	0.595
	over_sampling.BorderlineSMOTE	0.603
	over_sampling.RandomOverSampler	0.573
	over_sampling.SMOTE	0.579
	over_sampling.SVMSMOTE	0.584
Combine methods	combine.SMOTEENN	0.676
	combine.SMOTETomek	0.612

**Table A2:** The complete result of the feature adaptive base models.

Feature Subset	Dataset	FT_0_	Original AUC	FT_1_	Improved AUC	FT_0_	Original AUC	FT_1_	Improved AUC
		LR				SVM			
1	1	49	0.666	11	0.663	49	0.500	7	0.659
1	2	49	0.666	12	0.674	49	0.500	3	0.753
1	3	49	0.659	12	0.692	49	0.500	7	0.667
1	4	49	0.713	18	0.696	49	0.500	12	0.690
1	5	49	0.672	19	0.679	49	0.500	10	0.668
1	6	49	0.683	16	0.678	49	0.500	14	0.663
2	1	101	0.762	74	0.758	101	0.500	68	0.745
2	2	101	0.844	17	0.828	101	0.500	12	0.845
2	3	101	0.883	26	0.902	101	0.500	15	0.870
2	4	101	0.517	34	0.585	101	0.500	18	0.574
2	5	101	0.647	28	0.662	101	0.500	12	0.578
2	6	101	0.644	38	0.640	101	0.500	34	0.554
		RF				LGB			
1	1	49	0.693	4	0.764	49	0.726	8	0.749
1	2	49	0.679	4	0.807	49	0.723	7	0.753
1	3	49	0.669	4	0.863	49	0.773	6	0.878
1	4	49	0.715	18	0.682	49	0.721	16	0.682
1	5	49	0.699	11	0.700	49	0.705	8	0.699
1	6	49	0.703	7	0.691	49	0.701	12	0.724
2	1	101	0.757	67	0.760	101	0.771	64	0.769
2	2	101	0.769	9	0.808	101	0.818	18	0.848
2	3	101	0.866	11	0.887	101	0.860	14	0.886
2	4	101	0.582	41	0.602	101	0.609	33	0.591
2	5	101	0.707	21	0.697	101	0.682	32	0.676
2	6	101	0.677	21	0.610	101	0.689	26	0.681
		MLP				KNN			
1	1	49	0.666	6	0.782	49	0.687	7	0.767
1	2	49	0.645	8	0.788	49	0.685	8	0.766
1	3	49	0.661	14	0.849	49	0.691	6	0.862
1	4	49	0.696	12	0.714	49	0.704	18	0.714
1	5	49	0.500	8	0.698	49	0.677	10	0.706
1	6	49	0.699	13	0.725	49	0.678	10	0.711
2	1	101	0.750	68	0.749	101	0.756	77	0.746
2	2	101	0.772	12	0.854	101	0.782	13	0.803
2	3	101	0.850	21	0.886	101	0.904	15	0.901
2	4	101	0.660	26	0.653	101	0.585	25	0.587
2	5	101	0.681	38	0.684	101	0.715	14	0.653
2	6	101	0.518	28	0.644	101	0.707	23	0.650

a. Dataset division can be seen in Figure 3. b. FT_0_ represents the number of remaining features before the feature set is filtered by the adaptive feature selection method; FT_1_ represents the number of remaining features after the feature set is filtered by the adaptive feature selection method.

**Table A3:** The hyper parameters of all the models in three layers.

Category	Model	Hyper Parameters	Range of Adjustment	Final Values
Base model (first layer)	MLP	hidden_layer_sizes	1,2,3(layers)/(15,25,50)(nodes)	(11,20)
		solver	{lbfgs,sgd,adam}	‘adam’
		activation	{identity,LR,tanh,relu}	‘relu
		learning_rate_init	(0,0.1)	0.01
	KNN	n_neighbours	(2,30)	28
		algorithm	{ball_tree,kd_tree,brute,auto}	‘ball_tree’
		weights	{uniform,distance}	‘distance’
	LGB_1	num_leaves	(10,120)	80
		n_estimators	(50,200)	170
		min_child_weight	(0,30)	0
		learning_rate	(0.01,1)	0.52
		colsample_bytree	(0,1)	0.6
		subsample	(0,1)	0.1
	LGB_2	num_leaves	(10,120)	100
		n_estimators	(50,200)	200
		min_child_weight	(0,30)	0
		learning_rate	(0.01,1)	0.08
		colsample_bytree	(0,1)	0.8
		subsample	(0,1)	0.1
	RF	n_estimators	(50,200)	150
		min_samples_leaf	(1,10)	1
		max_depth	(3,8)	8
		max_features	(1,len(features)-1)	6
		min_impurity_decrease	(0,0.5)	0
Base model (second layer)	LGB_3	num_boost_round	{50,100,150,200}	300
		num_leaves	(2)	2
		max_depth	(0,3)	1
Meta model	LR	C	(0,1)	10 × 10^−10^
		penalty	{‘l1′,’l2′}	‘l2′
		solver	{‘newton-cg’, ‘lbfgs’, ‘liblinear’, ‘sag’, ‘saga’}	‘liblinear’
		max_iter	(0,10 × 10^7^)	10e7
		class_weight	‘balanced’	‘balanced’
